# Effective and Accurate Diagnosis of Subjective Cognitive Decline Based on Functional Connection and Graph Theory View

**DOI:** 10.3389/fnins.2020.577887

**Published:** 2020-09-29

**Authors:** Xiaowen Xu, Weikai Li, Mengling Tao, Zhongfeng Xie, Xin Gao, Ling Yue, Peijun Wang

**Affiliations:** ^1^Department of Medical Imaging, Tongji Hospital, Tongji University School of Medicine, Tongji University, Shanghai, China; ^2^College of Mathematics and Statistics, Chongqing Jiaotong University, Chongqing, China; ^3^Universal Medical Imaging Diagnostic Center, Shanghai, China; ^4^Department of Geriatric Psychiatry, Shanghai Mental Health Center, Shanghai Jiao Tong University School of Medicine, Shanghai, China; ^5^Alzheimer’s Disease and Related Disorders Center, Shanghai Jiao Tong University, Shanghai, China

**Keywords:** resting-state functional magnetic resonance imaging, functional connection, graph theory, multiple kernel learning, subjective cognitive impairment

## Abstract

Subjective cognitive decline (SCD) is considered the earliest preclinical stage of Alzheimer’s disease (AD) that precedes mild cognitive impairment (MCI). Effective and accurate diagnosis of SCD is crucial for early detection of and timely intervention in AD. In this study, brain functional connectome (i.e., functional connections and graph theory metrics) based on the resting-state functional magnetic resonance imaging (rs-fMRI) provided multiple information about brain networks and has been used to distinguish individuals with SCD from normal controls (NCs). The consensus connections and the discriminative nodal graph metrics selected by group least absolute shrinkage and selection operator (LASSO) mainly distributed in the prefrontal and frontal cortices and the subcortical regions corresponded to default mode network (DMN) and frontoparietal task control network. Nodal efficiency and nodal shortest path showed the most significant discriminative ability among the selected nodal graph metrics. Furthermore, the comparison results of topological attributes suggested that the brain network integration function was weakened and network segregation function was enhanced in SCD patients. Moreover, the combination of brain connectome information based on multiple kernel-support vector machine (MK-SVM) achieved the best classification performance with 83.33% accuracy, 90.00% sensitivity, and an area under the curve (AUC) of 0.927. The findings of this study provided a new perspective to combine machine learning methods with exploration of brain pathophysiological mechanisms in SCD and offered potential neuroimaging biomarkers for diagnosis of early-stage AD.

## Introduction

Alzheimer’s disease (AD) is an irreversible neurodegenerative disease, which is characterized by the continuous loss of neurons and cognitive function decline (Blennow et al., 2006). With the increasing aging population and failure of clinical trials for AD, early diagnosis and interventions for preclinical AD are urgent and critical. Subjective cognitive decline (SCD) is considered as the earliest preclinical stage of AD that precedes mild cognitive impairment (MCI) (Jessen et al., 2014). It refers to self-reported and persistent cognitive impairment (Jessen et al., 2020). Previous studies have indicated that patients with SCD were 4.5–6.5 times more likely to convert to MCI or AD than those without cognitive complaints (Reisberg et al., 2010; Tales et al., 2015). Therefore, SCD is a promising sign for early prediction and diagnosis of AD.

At present, a growing number of neuroimaging studies have suggested that patients with SCD show atrophy of gray matter volume, degeneration of white matter fiber structure, and reduction of spontaneous functional activity in the frontal, lateral temporal, and parietal cortices (Fan et al., 2018; Shu et al., 2018; Lin et al., 2019). The brain connectome, including functional connections and graph theory topological metrics, is based on functional network and has attracted increasing attention owing to the complex brain network mechanism and various diagnostic information (Biswal et al., 2010; delEtoile and Adeli, 2017; Filippi et al., 2018; Gao et al., 2020). In a series of resting-state functional brain network researches, Wang Y. et al. (2013) compared the functional connections among individuals with SCD, MCI, and normal controls (NCs) and found that the strength of the functional connection between the default mode network (DMN) and right hippocampus in the SCD group was stronger than that in the MCI group but weaker than that in the NCs. With respect to graph theory attributes, the results of Li et al. (2018) reported the SCD patients exhibited lower degree centrality in the inferior parietal region and higher degree centrality in the bilateral hippocampus and left fusiform gyrus than the NCs. However, most of these studies were conducted separately depending on brain network functional connections or some graph theory attributes, and the results were acquired based on group-level comparisons. Given the multimodal properties of the brain connectome, it is a challenge to identify the discriminative features and apply them to individual classifications of SCD.

To address these issues, machine learning approaches combining feature selection and classifier have been applied for early and accurate diagnosis of AD. For the multimodal properties of brain connectome, least absolute shrinkage and selection operator (LASSO) (Wee et al., 2014; Li et al., 2019) and Student’s *t*-test (Qiao et al., 2016) were used to identify the predominant features of the brain network. Considering that a brain node has a group of nodal graph metrics, the modified group-LASSO method is considered to be more suitable for feature selection of nodal graph metrics (Liu et al., 2019). Moreover, compared with the support vector machine (SVM), the results of previous studies have demonstrated that multiple kernel SVM combined with multimodal brain connectome can partially alleviate the high-dimensional curve of multiple features and achieve better classification performance in the diagnosis of MCI and AD (Dyrba et al., 2015; Xu et al., 2020).

Considering the potential advantages of machine learning methods, we intend to combine group-LASSO and multiple kernel-SVM (MK-SVM) methods to identify the most discriminative features of the brain connectome and conduct accurate identification of SCD patients from NCs. This study might provide valuable information for accurate diagnosis of SCD and to explore the pathophysiological mechanisms of the preclinical AD.

## Materials and Methods

### Participants

This study used a longitudinal case–control design based on data retrospectively selected from the China Longitudinal Aging Study (CLAS) (Xiao et al., 2013), which was a community-based study of all individuals with Han Chinese nationality and aged ≥ 60 years in Shanghai (Xiao et al., 2016). This study was approved by the ethics committee of Shanghai Mental Health Center, Shanghai Jiao Tong University School of Medicine. A total of 67 right-handed participants (including 22 SCD and 20 NCs) were enrolled in our study. All subjects had a data collection in 2012 and 2019. The data collected in 2012 included epidemiological investigation, neurologic examination, a battery of neuropsychological assessments, and three-dimensional T1-weighted imaging (3D-T1WI) scan. The neuropsychological assessments included the Mini Mental State Examination (MMSE) (Tsai et al., 2020), Montreal Cognitive Assessment (MoCA) (Hao et al., 2020), Auditory Verbal Learning Test (AVLT) (Zhao et al., 2015), Wechsler Intelligence Scale (WAIS) (Hulur et al., 2018), Geriatric Depression Scale (GDS) (Sawada et al., 2019), Self-Rating Anxiety Scale (SAS) (Bruck et al., 2019), and Subjective Cognitive Decline Self-administered Questionnaire (SCD-9) (Shirooka et al., 2018). Besides, in 2019, in addition to the above-mentioned data collection, the same group of follow-up subjects also performed resting-state functional magnetic resonance imaging (rs-fMRI) scan. Therefore, the research on functional brain network connectome in this study was only based on follow-up samples after 7 years.

The inclusion criteria of SCD were based on the conceptual framework proposed by the Subjective Cognitive Decline Initiative (SCD-I) (Jessen et al., 2014) that included the following: (a) onset age > 60 years; (b) self-perceived gradual decline in memory compared with a previous normal status within the last 5 years or as conformed by a close caregiver; (c) MMSE and MoCA scores within the normal range; and d) a Clinical Dementia Rating (CDR) score of 0 (Petersen et al., 2001). The NCs should be without cognitive decline and neuropsychological test scores within the normal range. The exclusion criteria were as follows: (a) neurology-related or cerebral vascular diseases (e.g., Parkinson’s disease, brain tumors, or intracranial aneurysms); (b) systemic diseases that could cause cognitive impairments (e.g., thyroid dysfunctions, syphilis, HIV, or severe anemia); (c) severe schizophrenia or mental retardation according to their medical records; (d) severe problems with vision, hearing, or speaking; and (e) the inability to participate actively in the neuropsychological evaluation.

### Data Acquisition

T1-weighted structural imaging and rs-fMRI scans were performed on each subject in the same session. All MRI data were acquired on a 3.0-T MR scanner (Magnetom^§^ Verio; Siemens, Munich, Germany) using a 32-channel head coil. All participants were instructed to keep their eyes closed (but not to fall asleep), try to think of nothing, and move as little as possible during the scan.

T1-weighted 3D high-resolution images were collected using magnetization-prepared rapid gradient echo (MP-RAGE) sequence with the following parameters: repetition time (TR) = 2,300 ms, echo time (TE) = 2.98 ms, flip angle = 9°, inversion time (TI) = 1,100 ms, matrix size = 240 × 256, field of view (FOV) = 240 × 256 mm, slice number = 192, thickness = 1.2 mm, and voxel size = 1.0 × 1.0 × 1.2 mm. The scan lasted 5 min and 12 s. Meanwhile, the parameters of the rs-fMRI protocol were collected as follows: axial slices, TR = 2,000 ms, TE = 30 ms, flip angle = 90°, FOV = 224 × 224 mm, matrix size = 64 × 64, number of slices = 31, thickness = 3.6 mm, and voxel size = 3.5 × 3.5 × 3.6 mm. Each scan collected 240 volumes with a scan time of 8 min and 6 s.

### Data Preprocessing

Data preprocessing was performed using Data Processing Assistant for Resting-State fMRI (DPARSF^1^) and Statistical Parametric Mapping (SPM12^2^). The first 10 time points were discarded to ensure stabilization of the initial signal and adaptation of participants to the environment. Timing correction to the last slice was conducted. Realignment for compensation of head-movement effects was achieved using a six-parameter rigid-body spatial transformation. All spatial movements was considered as < 3-mm displacement and < 3° rotation in any direction, and no participant was excluded. Next, rs-fMRI images were co-registered to the high-resolution 3D-T1 structural images. Normalization of 3D-T1 structural MRI images to the Montreal Neurological Institute (MNI) space was performed by non-linear warping based on Diffeomorphic Anatomical Registration Through Exponentiated Lie Algebra (DARTEL). Then, rs-fMRI images were spatially normalized to the MNI space using the same parameters derived from the normalization of structural images and simultaneously resampled into 3-mm isotropic voxels. All normalized fMRI images were smoothed with a 6-mm, full-width at half maximum Gaussian kernel. Linear detrending and band-pass filtering at 0.01–0.1 Hz were carried out to control low-frequency drift and high-frequency physiological noise. Finally, nuisance covariates were regressed out, including the Friston 24-motion parameter model (six head-motion parameters, six head-motion parameters one time point before, and the 12 corresponding squared items); global mean; white matter; and cerebrospinal fluid signals.

### Brain Network Construction

The average time series within each region based on the automated anatomical labeling (AAL) atlas were separately extracted to construct the connectivity brain network (Tzourio-Mazoyer et al., 2002). The Pearson correlation coefficients of all pairs of 90 regions of interest (ROIs) were applied separately to define the edges of functional connections. Thus, the functional connectivity matrix (adjacency matrix) was constructed. The final functional connection networks produced N ^∗^ (N - 1)/2 edges, where N corresponded to the number of nodes in the networks. Considering the ambiguous interpretation of negative correlations, we restricted the analysis to positive correlations and set the negative correlation coefficients as zero. A thresholding method rely on network sparsity was conducted to discard the less significant connections and retain the topological properties of graph theory by setting an appropriate threshold for network sparsity (Dai et al., 2019). Sparsity thresholds (ranging from 0.02 to 0.5, with steps of 0.01) were set to acquire a binary undirected network. In order to avoid the influence of sparsity threshold on graph theory, the area under the curve (AUC) is adopted as the feature. It represents the sum of graph theory attributes of brain networks obtained under different sparsity thresholds. Therefore, the AUC, the sum value of 49 values of the corresponding node attributes, is used as input for the node attribute to train the classifier.

### Computation of Graph Metrics

Based on the binary undirected matrices, we analyzed the graph theory topological properties of the functional brain network by Graph Theoretical Network Analysis Toolbox (GRETNA^3^) based on Statistical Parametric Mapping (SPM8^4^) with MATLAB R2013b. As shown in Table 1, global and nodal topological metrics were applied to characterize the different patterns of connections in the brain network (Wang et al., 2015). The modularity (*Q*) of a brain network quantified the efficiency of segmenting a brain network into modules (Newman, 2006). The greedy optimization algorithm was used as follows:

**TABLE 1:** Global and nodal graph metrics of the brain connectome.

**Global graph metrics**	**Nodal graph metrics**
Clustering coefficient (C_*p*_)	Betweenness centrality
Characteristic path length (L_*p*_)	Degree centrality
Normalized clustering coefficient (γ)	Nodal clustering coefficient
Normalized characteristic path length (λ)	Nodal efficiency
Small-world (σ)	Nodal local efficiency
Global efficiency, E_*global*_	Nodal shortest path
Modularity (Q)	

$$Q=\sum_{i=1}^{N_{m}}\left[l_{i}/L-{\left(d_{i}/2\,L\right)}^{2}\right]$$

where *N*_*m*_ is the number of modules, *L* is the total number of edges in the brain network, *l*_*i*_ represents the number of within-module edges in the module *i*, and *d*_*i*_ is the sum of the linked edges at each node within the module *i*. Modified greedy optimization was used to detect the modular structure (Newman, 2004). Moreover, according to the definition of “hubs” (Rubinov and Sporns, 2010), we identified the top 5% of brain regions with the greatest weight in both SCD patients and NCs.

### Statistical Analyses

In terms of demographics and clinical characteristics, two-sample Student’s *t*-tests were performed except for sex (which was tested by the chi-square test). The clinical and demographic data of the participants are summarized in Table 2. *P* < 0.05 indicated a significant difference in demographic data. Comparison of graph theory metrics between SCD patients and NCs were carried out based on two-sample Student’s *t*-tests. A procedure to ascertain the false discovery rate was performed to further correct for multiple comparisons. *P* < 0.05 indicated a significant difference. In addition, for the functional connections, we selected connections by based on Student’s *t*-tests (*P*-value < 0.05). As the selected connections in each inner loop might be different, we identified the consensus connections for the classification model in each inner leave-one-out cross-validation (LOOCV) loop to ensure that the selected connections in each inner loop might be consistent.

**TABLE 2:** Demographics and clinical characteristics of patients with subjective cognitive decline (SCD) and normal controls (NCs).

**Characteristic/test**	**SCD**	**NC**	**T/χ^2^/Z**	***P***
Age (years)	*74*. 0 ±*5*.*6*	71.8 ± 2.9	1.67^a^	0.11
Sex (F/M)	14/8	6/14	5.31^b^	0.05
Education	10.1 ± 2.0	10.4 ± 3.0	0.00^c^	1.00
MMSE	27.6 ± 1.8	28.2 ± 1.6	−1.13^c^	0.26
MoCA	23.6 ± 3.9	24.1 ± 3.8	−0.48^c^	0.63
AVLT-Immediate Recall	5.5 ± 1.9	4.8 ± 1.5	−0.98^c^	0.33
AVLT-Short Delayed Recall	8.1 ± 2.6	8.2 ± 2.1	−0.14^a^	0.89
AVLT-Long Delayed Recall	30.9 ± 7.7	33.2 ± 7.6	−1.00^c^	0.32
AVLT-Recognition	10.2 ± 3.1	11.2 ± 3.0	−0.95^c^	0.34
Digit Span forward	8.0 ± 2.3	9.0 ± 2.0	−1.61^a^	0.11
Digit Span backward	5.3 ± 1.9	6.2 ± 2.3	−1.35^a^	0.19
WAIS picture completion	10.1 ± 2.7	11.9 ± 3.6	−1.76^a^	0.09
WAIS block design	27.2 ± 7.3	30.4 ± 6.7	−1.53^c^	0.13
GDS	6.5 ± 5.5	2.8 ± 2.6	−2.65^c^	0.01*
SAS	26.4 ± 4.5	23.9 ± 4.4	−1.93^c^	0.05
SCD-9	3.8 ± 1.9	2.4 ± 2.0	0.58^a^	0.03*

MMSE, Mini Mental State Examination; MoCA, Montreal Cognitive Assessment; AVLT, Auditory Verbal Learning Test; WAIS, Wechsler Intelligence Scale; GDS, Geriatric Depression Scale; SAS, Self-Rating Anxiety Scale; SCD-9, Subjective Cognitive Decline Self-administered Questionnaire. Data are presented as the mean ± SD. *P < 0.05 indicates significant differences between the groups. ^*a*^T value was obtained by using the two-sample t-test. ^*b*^*χ*^2^value was obtained using the chi-square test. ^*c*^Z value obtained by using the rank-sum test.

### Feature Selection

**FIGURE 1:**
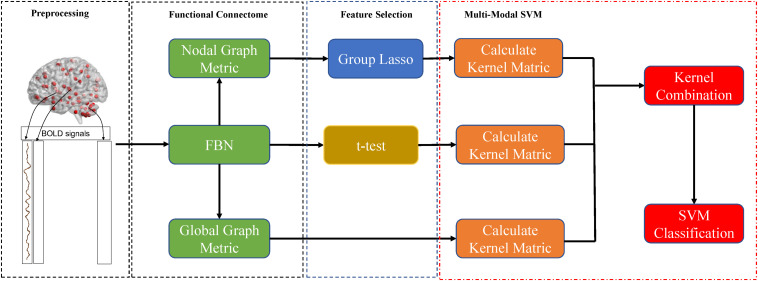
Procedures of data processing and classification in the present study.

As mentioned above, the brain was divided into 90 nodes based on the AAL90, and each node corresponded to six local graph metrics (Figure 1). Thus, the nodal graph metrics naturally have a group topology; i.e., a node corresponds to a group of node-graph theoretical attributes. Given the natural group attributes, we used group-LASSO as the feature-selection scheme for nodal graph metrics.

$$m\,i\,n_{w}\,\sum_{i=1}^{n}l\,o\,g\,\left(1+e\,x\,p\,\left(-y_{i}\times \left(\sum_{j=1}^{n\,R\,O\,I}\sum_{k=1}^{6}w_{\left(j,k\right)}\,x_{\left(j,k\right)}+c\right)\right)\right)+\lambda \,\sum_{i}^{n\,R\,O\,I}\left|\sum_{k}^{6}w_{j\,k}^{2}\right|,$$

where *y*_*i*_ is the label of the *i*-th participant, and *w* and *x*_*j,k*_ are the weight and value of the *j*-th ROI and *k*-th nodal graph metric, respectively. It is notable that *x*_*j,k*_ is normalized by Fisher Z-transformation to avoid scale imbalance. We used the SLEP toolbox^5^ to calculate *w* with a default setting of λ=1.

In present study, we follow the most commonly used nested cross-validation scheme to evaluate the performance of the proposed multiple kernel method (Varma and Simon, 2006). Thus, the features were selected for each iteration of the LOOCV procedure. As the selected connections in each inner loop might be different, we further identified the consensus connections for discovering the biomarker toward SCD identification.

### Classification

To overcome the limitation of high-dimensional curve and small number, we used MK-SVM with the kernel combination trick in our study for information combination to partially alleviate the annihilation of high-dimensional information to low-dimensional information. MK-SVM was carried out listed as follows.

In the present study, there are *n* training samples of functional connections and graph metrics. ${\text{x}}_{\text{i}}^{1},{\text{x}}_{\text{i}}^{2},{\text{x}}_{\text{i}}^{3},$ and y_i_ ∈ {1,−1} represent the discriminative connection, global graph metrics, nodal graph metrics, and its corresponding class label of the *i*-th sample, respectively. MK-SVM calculates the problem as follows:

$$\min_{\text{W}}\frac{1}{2}\,\sum_{m=1}^{3}\beta_{m}\,{||w^{m}||}^{2}+C\,\sum_{i=1}^{n}\xi_{i}$$

$$\text{s}.\text{t}.y_{i}\,\left(\sum_{m=1}^{3}\beta_{m}\,{\left(w^{m}\right)}^{T}\,\phi^{m}\,\left(x_{i}^{m}\right)+b\right)\geq 1-\xi_{i}$$

$$\xi_{i}\geq 0,i=1,2,\ldots ,n$$

where ϕ^*m*^ is mapping from the original space to the reproducing kernel Hilbert space (RKHS), *w^m^* represents the normal vector of the hyperplane in RKHS, and β_*m*_ denotes the corresponding combining weight on the *m*-th modality. Then, the dual form of MK-SVM can be represented as follows:

$$\max_{\alpha }\,\sum_{i=1}^{n}\alpha_{i}-\frac{1}{2}\,\sum_{i,j}\alpha_{i}\,\alpha_{j}\,y_{i}\,y_{j}\,\sum_{m=1}^{3}\beta_{m}\,k^{m}\,\left(x_{i}^{m},y_{i}^{m}\right)$$

$$\text{s}.\text{t}.\sum_{i=1}^{n}\alpha_{i}\,y_{i}=0$$

$$0\leq \alpha_{i}\leq C,i=1,2,\ldots ,n$$

where $k^{m}\,\left(x_{i}^{m},y_{i}^{m}\right)=\phi^{m}\,{\left(x_{i}^{m}\right)}^{T}\,\phi^{m}\,\left(x_{j}^{m}\right)$ and is the kernel matrix on the *m*-th modality. After we trained the model, we tested the new samples *x* = {*x*_1_,*x*_2_,…,*x*_*M*_}. The kernel between the new test sample and *i*-th training sample on the *m*-th modality is defined as $k^{m}\,\left(x_{i}^{m},x^{m}\right)=\phi^{m}\,{\left(x_{i}^{m}\right)}^{T}\,\phi^{m}\,\left(x^{m}\right)$. In the end, the predictive level based on MK-SVM was calculated as follows:

$$f\,\left(x_{1},x_{2},\ldots ,x_{m}\right)=\text{sign}\,\left(\sum_{i=1}^{n}y_{i}\,\alpha_{i}\,\sum_{m=1}^{3}\beta_{m}\,k^{m}\,\left(x_{i}^{m},x^{m}\right)+b\right)$$

The proposed MK-SVM in this study could be considered an innovative multiple kernel learning method, because β_*m*_ is selected based on the cross-validation scheme on the grid-searching space with constraints ∑_m_β_*m*_ = 1. The range of c was 2^−5^ to 2^5^. All data processing and classification procedures used in our study are shown in Figure 1. To deal with the small sample size, we used the LOOCV strategy to verify the performance of the methods, in which only one subject is left out for testing, while the others are used to train the models and obtain the optimal parameters. For the choice of optimal parameters, an inner LOOCV was conducted on the training data by using a grid-search strategy.

## Results

### Demographics and Clinical Characteristics

The demographics and clinical characteristics of all subjects are summarized in Table 2. The scores of GDS and SCD-9 in the SCD group were significantly higher than those in the NCs (*P* < 0.05), which indicated SCD patients show higher depression rate than that of NCs. There were no significant differences with respect to age, sex, education, and other scales.

### Consensus Connections of Brain Network

**FIGURE 2:**
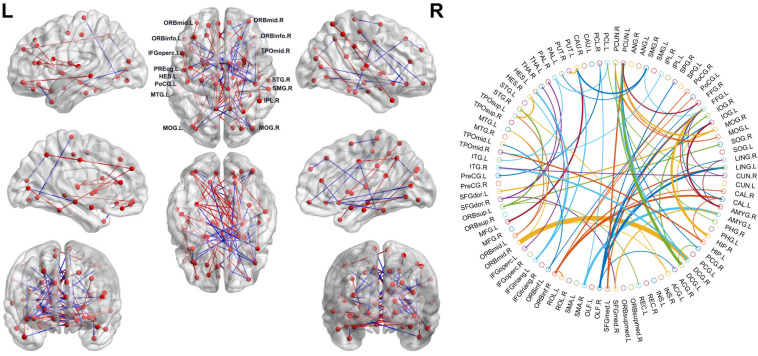
Left: The consensus connections selected by leave-one-out cross-validation (LOOCV). The connections were mapped on the ICBM 152 template with the BrainNetViewer package (http://nitrc.org/projects/bnv/). Blue and red represent the decrease and increase of functional connection weight of subjective cognitive decline (SCD) groups, respectively. Right: The consensus connections of functional brain network selected by LOOCV in SCD and normal control (NC) groups based on AAL90. The thickness of an arc in the circle indicates the discriminative power of an edge, which is inversely proportional to the estimated *P*-values. The colors were randomly generated to differentiate regions of interests (ROIs). The figure was conducted with a MATLAB function, circularGraph, shared by Paul Kassebaum. (http://www.mathworks.com/matlabcentral/fileexchange/48576-circulargraph).

As mentioned above, we selected the consensus connections with P < 0.05 in each loop. A total of 72 consensus connections are shown in Figure 2. All consensus connections had both increased and decreased functional connections in SCD patients. We projected them into the corresponding subnetworks and found that most consensus connections were mainly distributed in the DMN and frontoparietal task control network. Because degree attribute represents the number of functional connectivity edges of nodes, in this study, we further detected the distribution of functional connections across the whole brain by using the nodal degree. The results indicated that the brain regions with the highest degree were mainly distributed in the frontal and prefrontal cortices and the subcortical areas. It is notable that the decreased degree values of SCD patients in these regions suggest the reduced number of functional connections.

### Global Graph Metrics of the Functional Brain Connectome

**FIGURE 3:**
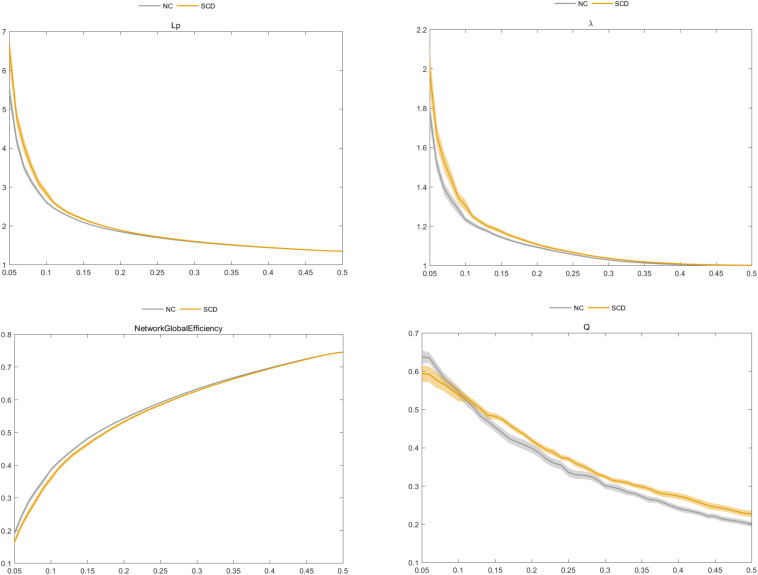
Comparison of characteristic path length (L_p_), normalized characteristic path length λ, global efficiency (E_global_), and modularity (Q) between the subjective cognitive decline (SCD) and normal control (NC) groups.

We found that the SCD and NC groups met the topological attributes of “small-world.” That is, the brain networks of SCD and NCs had larger C_p_ and almost identical L_p_ than the matched random networks. With the increase in connection density, the value of C_p_ increased, but values of L_p_, γ, λ, and σ decreased in the SCD and NC groups. Statistical analysis showed that the E_global_ value of SCD patients was lower than that of NCs, while the values of L_p_, λ, and Q in the SCD group were higher than those in NCs (*P* < 0.05) (Figure 3).

### Nodal Graph Metrics of the Functional Brain Connectome

**FIGURE 4:**
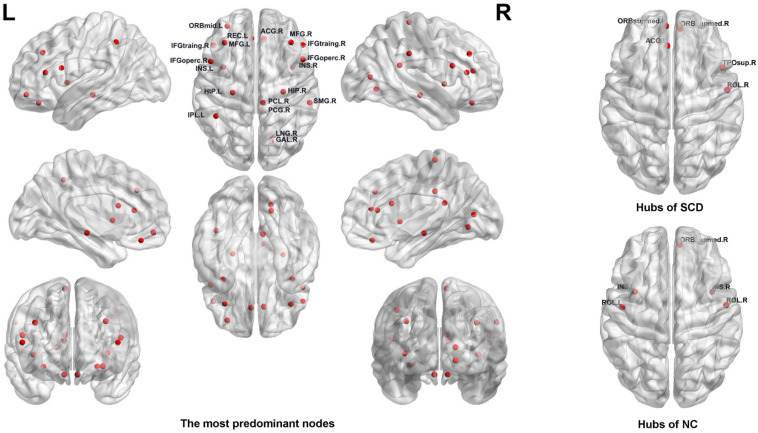
**Left**: The most predominant nodes for discriminating subjective cognitive decline (SCD) from normal control (NC) groups selected by group least absolute shrinkage and selection operator (LASSO). **Right**: Hub nodes of SCD and NC groups in the brain network.

For the nodal graph metrics of the functional brain connectome, we identified the most predominant brain regions and the most discriminative nodal graph metrics, which were selected by group-LASSO. First, our results showed the top 20 most predominant brain nodes with the most significant differences in nodal graph metrics. As shown in Table 3 and Figure 4, most of the predominant brain regions were distributed in the prefrontal and parietal cortices and the subcortical regions. In particular, the insula is attributed to subcortical region exhibited four nodal graph metrics with significant differences. The prefrontal cortex, including the middle frontal gyrus, inferior frontal gyrus, and gyrus rectus, accounts for the maximum number of the top 20 most predominant brain nodes.

**TABLE 3:** Top 20 predominant brain nodes with the significant differences in nodal graph metrics.

**AAL number**	**Corresponding brain regions**	**Number of nodal metrics**	**Anatomical classification**
30	Insula_R	4	Subcortical
70	Paracentral_Lobule_R	3	Parietal
29	Insula_L	2	Subcortical
32	Cingulum_Ant_R	1	Prefrontal
38	Hippocampus_R	1	Temporal
7	Frontal_Mid_L	1	Prefrontal
8	Frontal_Mid_R	1	Prefrontal
9	Frontal_Mid_Orb_L	1	Prefrontal
11	Frontal_Inf_Oper_L	1	Prefrontal
12	Frontal_Inf_Oper_R	1	Prefrontal
13	Frontal_Inf_Tri_L	1	Prefrontal
14	Frontal_Inf_Tri_R	1	Prefrontal
27	Rectus_L	1	Prefrontal
28	Rectus_R	1	Prefrontal
36	Cingulum_Post_R	1	Parietal
37	Hippocampus_L	1	Temporal
44	Calcarine_R	1	Occipital
48	Lingual_R	1	Occipital
61	Parietal_Inf_L	1	Parietal
64	SupraMarginal_R	1	Parietal

*AAL, automated anatomical labeling atlas.*

Moreover, we found that the nodal graph theory showed the maximum discriminative ability, selected by group-LASSO (Figure 4). Table 4 shows the top 20 nodal graph topological features with the largest discriminative ability. Our results indicated that the nodal graph metric with the most significant difference was nodal efficiency, and the corresponding brain region was the insula attributed to the subcortical region. Meanwhile, the nodal shortest path, which accounts for the largest proportion of the top 20 nodal graph metrics and most of the corresponding brain regions, was distributed in the prefrontal and parietal cortices.

**TABLE 4:** Top 20 nodal graph theory features with maximum discriminative ability selected by group-LASSO.

**Nodal graph measures**	**AAL number**	**Corresponding brain regions**	**Anatomical classification**
Nodal efficiency	30	Insula_R	Subcortical
Nodal clustering coefficient	70	Paracentral_Lobule_R	Parietal
Nodal shortest path	11	Frontal_Inf_Oper_L	Prefrontal
Nodal shortest path	13	Frontal_Inf_Tri_L	Prefrontal
Nodal shortest path	29	Insula_L	Subcortical
Betweenness centrality	70	Paracentral_Lobule_R	Parietal
Nodal efficiency	29	Insula_L	Subcortical
Nodal shortest path	30	Insula_R	Subcortical
Betweenness centrality	38	Hippocampus_R	Temporal
Nodal clustering coefficient	36	Cingulum_Post_R	Parietal
Nodal shortest path	61	Parietal_Inf_L	Parietal
Nodal local efficiency	70	Paracentral_Lobule_R	Parietal
Betweenness centrality	28	Rectus_R	Prefrontal
Degree centrality	27	Rectus_L	Prefrontal
Nodal shortest path	14	Frontal_Inf_Tri_R	Prefrontal
Nodal shortest path	8	Frontal_Mid_R	Prefrontal
Nodal shortest path	9	Frontal_Mid_Orb_L	Prefrontal
Nodal shortest path	17	Rolandic_Oper_L	Frontal
Betweenness centrality	32	Cingulum_Ant_R	Prefrontal
Betweenness centrality	30	Insula_R	Subcortical

*AAL, automated anatomical labeling atlas; LASSO, least absolute shrinkage and selection operator.*

In addition, our results identified the hub nodes of SCD patients and NCs. As shown in Table 5 and Figure 4, the common hubs of SCD patients and NCs were located mainly in the frontal and prefrontal cortices. More importantly, some hub nodes such as the temporal pole and anterior cingulate and paracingulate gyri were present only in SCD patients. Further, there were also some hub nodes such as bilateral insulars that only existed in NCs.

**TABLE 5:** Hubs of SCD and NCs defined with the degree.

	**AAL number**	**Corresponding brain regions**	**Anatomical classification**
SCD	25	Frontal_Mid_Orb_L	Prefrontal
	26	Frontal_Mid_Orb_R	Prefrontal
	84	Temporal_Pole_Sup_R	Temporal
	18	Rolandic_Oper_R	Frontal
	31	Cingulum_Ant_L	Prefrontal
NC	26	Frontal_Mid_Orb_R	Prefrontal
	17	Rolandic_Oper_L	Frontal
	29	Insula_L	Subcortical
	30	Insula_R	Subcortical
	18	Rolandic_Oper_R	Frontal

*AAL, the automated anatomical labeling atlas; SCD, subjective cognitive decline; NC, normal control.*

### Classification

**FIGURE 5:**
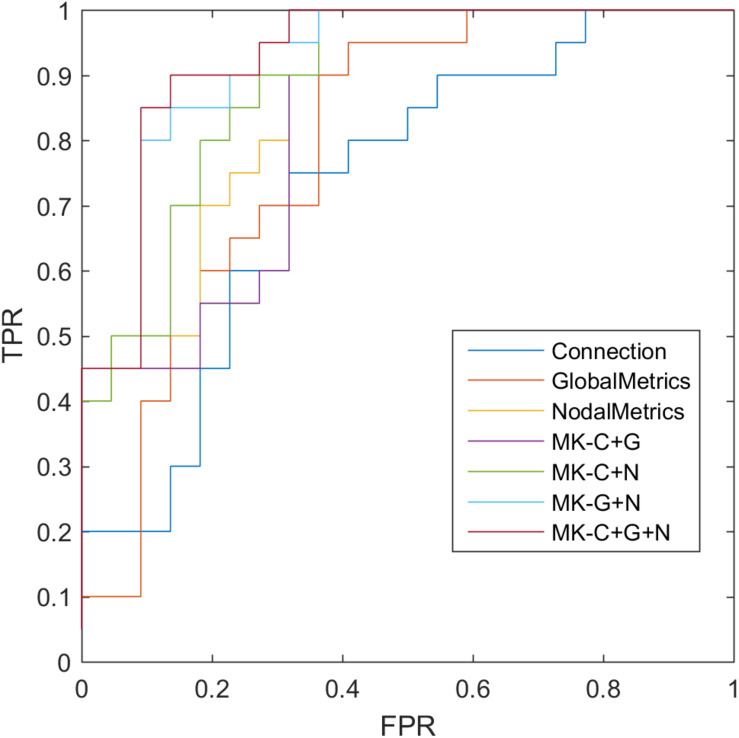
Receiver operating characteristic (ROC) of classification based on different features. C, connection; G, global metrics; N, nodal metrics; MK, multiple kernel; FPR, false-positive rate; TPR, true-positive rate.

After the selection of discriminative features of brain network connectome, MK-SVM was used to combine the brain connectome information. The performance of classification with different brain network features was evaluated based on the values of accuracy, sensitivity, and specificity, as follows:

$$A\,c\,c\,u\,r\,a\,c\,y=\frac{T\,P+T\,N}{T\,P+F\,P+T\,N+F\,N},$$

$$S\,e\,n\,s\,i\,t\,i\,v\,i\,t\,y=\frac{T\,P}{T\,P+F\,N},$$

$$S\,p\,e\,c\,i\,f\,i\,c\,i\,t\,y=\frac{T\,N}{T\,N+F\,P},$$

where TP, TN, FP, and FN denote the number of true-positive, true-negative, false-positive, and false-negative values, respectively. The area under the receiver operating characteristic curve (AUC) was calculated as a performance measure for binary classification of SCD and NCs. In particular, LOOCV was employed in this study because of the small sample size, which provided an optimistic estimate of the classification accuracy, as all except one of the subjects was used to train the classifier. For other approaches such as the k-fold cross-validation, only N - k (where N is the total number of participants in the dataset) participants were included during the training process, resulting in poorer performance given the small dataset. For the functional connections (C), global metrics (G), and nodal metrics (N) of the brain network, we obtained AUCs of 0.728, 0.793, and 0.865, respectively (Table 6 and Figure 5). Moreover, after combining functional connections and global metrics (C + G), functional connections and nodal metrics (C + N), and global metrics and nodal metrics (G + N), the AUCs of classification were 0.836, 0.888, and 0.918, respectively. Finally, combinations of all brain network connectome features based on MK-SVM achieved the best classification performance with 83.33% accuracy and 90.00% sensitivity and an AUC of 0.927. To investigate the significance of model performance improvement, differences between various AUCs were compared by using a Delong test (DeLong et al., 1988). The statistical tests compared with MK_SVM and the single modal methods were two sided, and *P*-values less than 0.05 (*P* = 0.00654, global metrics vs. MK_C + G + N; P = 0.027, connection vs. MK_C + G + N, P = 0.014; and nodal metrics vs. MK_C + G + N) indicated statistical significance, while the *P*-value statistical tests compared with MK_C + G + N with two model methods are less significant, with *P*-values of 0.035 (MK_C + G vs. MK_C + G + N), 0.031 (MK_N + G vs. MK_C + G + N), and 0.027 (MK_C + N vs. MK_C + G + N).

**TABLE 6:** The evaluation of classification performance corresponding to different functional connectome features.

**Methods**	**Accuracy (%)**	**Sensitivity (%)**	**Specificity (%)**	**AUC**
Connection (C)	64.29	50.00	77.23	0.728
Global Metrics (G)	69.05	65.00	72.73	0.793
Nodal Metrics (N)	73.81	80.00	68.18	0.865
MK_C + G	71.43	75.00	68.18	0.836
MK_C + N	78.57	85.00	72.73	0.888
MK_G + N	80.95	85.00	77.27	0.918
MK_C + G + N	83.33	90.00	77.27	0.927

*MK-SVM, multiple kernel-support vector machine.*

## Discussion

In this study, we applied machine learning methods with functional connections and topological metrics to investigate the most discriminative features of the brain connectome, accurately diagnose SCD, and achieve meaningful results. First, our results suggested that the consensus connections selected by *t*-test and the discriminative nodal graph metrics selected by group-LASSO were mainly distributed in the prefrontal and frontal cortices and the subcortical regions, which corresponds to the DMN and frontoparietal task control network. Second, the comparison results of topological attributes suggested that the brain network integration function was weakened and segregation function was enhanced in SCD patients. Moreover, the combination of brain connectome information based on MK-SVM effectively improved the classification performance.

From the distribution of both consensus connections and nodal graph metrics, we found that the predominant brain regions in SCD patients with significant differences were mainly distributed in the prefrontal cortex (bilateral middle frontal gyrus, bilateral inferior frontal gyrus, and bilateral gyrus rectus); parietal cortex (left inferior parietal and right supramarginal gyrus); and subcortical regions (bilateral insula). Previous studies have also found abnormalities in some of these brain regions mentioned above. For instance, Yan et al. (2019) identified the most discriminating brain regions by the methods of elastic net, LASSO, and Fisher score, which were mainly located in the medial prefrontal cortex (mPFC) and subcortical structures. Chen et al. (2020) found that the nodal global efficiency and nodal local efficiency were increased in SCD patients, which were mainly located in the frontal, medial temporal, and parietal cortices (Chen et al., 2020). Multiple evidences have suggested that self-referential processing is mediated by cortical midline structures such as the ventromedial and dorsomedial prefrontal cortex and the anterior and posterior cingulate cortex (Yasuno et al., 2015). Nevertheless, the results of SCD studies suggested that individuals with preclinical AD were more likely to present functional network abnormalities in the prefrontal cortex than MCI patients in previous studies, who showed the most significant differences of brain connectome mainly distributed in the medial temporal lobe (Xu et al., 2020). Compared with the distribution of brain regions with significant differences (e.g., medial temporal lobe) in MCI patients as detected in previous studies, the results suggested that patients with SCD were more likely to show changes to the functional networks in the prefrontal cortex. Furthermore, from the perspective of subnetworks, most of these brain regions mentioned above were distributed in the DMN and frontoparietal task control, which is consistent with previous studies. Our results not only showed that DMN is considered the most vulnerable functional subnetwork in the early stage of AD (Meskaldji et al., 2016; Xie et al., 2019) but also showed the repeatability and verifiability of the proposed methods, which is an important contribution of our research.

In addition, we explored the topological features of the nodal graph with the largest discriminative ability. Our results indicated that nodal efficiency and nodal shortest path had the most significant discriminative ability among the selected nodal graph metrics. The changes of nodal graph metrics have also been mentioned in previous studies (Li et al., 2018). Meanwhile, our findings suggested that the corresponding brain regions with the most discriminative nodal graph metrics overlapped with the hub nodes found in SCD patients. Therefore, our results emphasize the importance of analyzing the brain connectome for early discrimination of SCD patients from NCs.

With respect to the altered pattern of functional brain connectome in SCD patients, our results indicated that patients with SCD and NC groups fit the global topological attributes of “small-world.” That is, the brain network supported rapid and real-time information integration between different brain regions and could maximize the efficiency of active information processing between brain regions with minimum cost (Liao et al., 2017). However, the increase of L_p_ and λ values and the decrease of E_global_ value suggested the decrease of brain network integration function in SCD patients; i.e., the global information communication and transmission abilities of the whole brain network reduced. At the same time, the increase of Q value indicated that the brain network of SCD patients tended to be more modular, and the network segregation function of information communication and transmission between local information in the brain network was enhanced (Wang J. et al., 2013). This compensatory change in the local function of the brain network may be used to explain the mild neuronal injury in SCD but with clinically normal residual cognitive manifestation (Sperling et al., 2011; Li et al., 2018).

It is a challenge to identify objective and accurate neuroimaging biomarkers and apply them to the individual classification of SCD. In our study, considering a brain node corresponds to a group of nodal topological attributes, we adopted the modified group-LASSO algorithm to select the predominant brain regions and the most discriminative nodal graph metrics. Compared with Student’s *t*-test and LASSO, the proposed group-LASSO is more suitable for the feature selection of nodal graph metrics. It can reserve the most discriminative features while alleviating redundancy of information. Finally, MK-SVM was used to combine the multimodal features of the brain connectome, which partially alleviated the high-dimensional curve of multiple features and achieved the best classification performance with 83.33% accuracy, 90.00% sensitivity, and an AUC of 0.927. Compared with previous studies performed by Yan et al. (accuracy: 79.23%; Yan et al., 2019) and Chen et al. (accuracy: 80.24%; Chen et al., 2020), our results suggested the feature selection and combination of multimodal brain connections could improve the classification performance of SCD. Furthermore, differences between various AUCs were compared by using a Delong test, which demonstrated that the proposed method combined with multimodal features of brain network connectome significantly outperformed the single modality based on current samples.

### Limitations and Future Directions

Our study has some limitations. First, the sample size of the study was small. Although our method achieved a good classification performance based on the current samples, we look forward to expanding the sample size in future research to further validate the robustness and generalizability of our proposed method. In addition, our approach has only been validated on single-center data, but a large amount of data are currently from multicenter sources. Therefore, in the future work, the performance of the proposed method needs to be further validated based on multicenter data. The second is the combination of multimodal diagnostic information. In future research, we can classify and explore the pathological mechanisms of SCD by combining multimodal diagnostic information by means of structural MRI, PET-MRI, and blood biomarkers. Moreover, our results suggested that SCD patients showed a higher rate of depression than the NCs. Previous studies have also shown that SCD is usually associated with mild symptoms of depression (Hill et al., 2016; Jessen et al., 2020). It might co-occur with SCD due to a common underlying cause, or as a result of SCD itself. Nevertheless, the specific mechanism between SCD and depression still needs to be further verified.

## Conclusion

Our results showed that the discriminative brain connectome features were mainly distributed in the prefrontal and frontal cortices and the subcortical regions, which corresponded to the DMN and frontoparietal task control network. The comparison results of topological attributes indicated that the brain network integration function was weakened and segregation function was enhanced in SCD patients. Moreover, the combination of brain connectome information based on MK-SVM greatly improved the classification performance. The findings of this study might provide valuable information for accurate diagnosis of preclinical AD and to better understand its pathological mechanisms, which might provide a crucial opportunity for postponing and even preventing the progression of this disease.

## Data Availability Statement

All datasets generated for this study are included in the article/supplementary material.

## Ethics Statement

The studies involving human participants were reviewed and approved by the Institution’s Ethical Committee of Shanghai Mental Health Center, Shanghai Jiao Tong University School of Medicine. The patients/participants provided their written informed consent to participate in this study. Written informed consent was obtained from the individual(s) for the publication of any potentially identifiable images or data included in this article.

## Author Contributions

XX and WL designed the study and drafted the manuscript. LY, MT, and ZX acquired the MRI data and diagnosis of the subjects. XX, WL, and LY analyzed and interpreted the results of the data. XG and PW revised the manuscript. All authors approved the final manuscript.

## Conflict of Interest

The authors declare that the research was conducted in the absence of any commercial or financial relationships that could be construed as a potential conflict of interest.

## Footnotes

1 http://restfmri.net/forum/index.php

2 http://www.fil.ion.ucl.ac.uk/spm

3 www.nitrc.org/projects/gretna/

4 www.fil.ion.ucl.ac.uk/spm

5 www.yelab.net/software/SLEP
